# Comorbidities in children with Internet Addiction Disorder (IAD)

**DOI:** 10.1192/j.eurpsy.2022.2154

**Published:** 2022-09-01

**Authors:** T. Nadiradze, S. Bakhtadze, N. Khachapuridze

**Affiliations:** 1 G. Zhvania Academic Clinic of Pediatry, Tbilisi State Medical University, Department Of Pediatric Neurology, Tbilisi, Georgia; 2 G. Zhvania Academic Clinic of Pediatry, Tbilisi State Medical University, Department Of Pediatric Neurology, Tbilisi, Georgia

**Keywords:** Depression, fatigue, internet addiction

## Abstract

**Introduction:**

Internet addiction disorder (IAD) is characterized by an individual’s inability to control his/her Internet use, which may result in marked distress and functional impairment. Systematic reviews show that excessive screen-time is negatively associated with well-being and positively associated with reduced quality of life in young people. There is growing evidence that IAD is related to comorbidities such as depression but relatively little is known about fatigue in adolescents with IAD.

**Objectives:**

Accumulating evidence suggests that fatigue is a central component of IAD. Depression is also related to IAD. However, there is a lack of evidence regarding whether there is a strong correlation between the severity of IAD and the rate of depression. Our objectives were to describe depression and fatigue in adolescents diagnosed with IAD.

**Methods:**

Study included 94 participants with IAD and 88 controls, all aged 12–17 years. Depression was assessed by the Beck Depression Inventory Scale (Georgian version), and fatigue by the Pediatric Quality of Life Initiative (Georgian version) multidimensional fatigue scale.

**Results:**

Adolescents with severe IAD are 5.63 times more likely to show symptoms of moderate or severe depression than children with mild or moderate Internet addiction. Those with severe IAD showed 6.62 times more cognitive fatigue, 7.81 times higher sleep/rest fatigue and 11.11 times higher general fatigue than children with mild and moderate Internet addiction.

**Conclusions:**

IAD can lead to depression and fatigue, which can affect adolescent’s psychological and social well-being. Mechanisms for prevention and ongoing support are needed for adolescents and their families.

**Disclosure:**

No significant relationships.

